# Sensory Loss and the Healthcare System: Outcomes and Navigation

**DOI:** 10.1093/geroni/igaa057.2888

**Published:** 2020-12-16

**Authors:** Nicholas Reed, Charlotte Yeh

## Abstract

Communication is fundamental to patient-centered care. However, sensory impairment limits communication among older adults. Specifically, hearing impairment strains communication via degraded auditory encoding while vision impairment distresses ability to read and interpret visual cues. The presence of dual sensory impairment, defined as concurrent hearing and vision impairment, may exacerbate these effects. The potential consequence s of age-related sensory loss on health care interactions and outcomes are beginning to surface in epidemiologic studies demonstrating poorer patient-provider communication, higher medical expenditures, increased risk of 30-day readmission, and longer length of stay when compared to individuals without sensory loss. Importantly, these associations may be amenable to intervention via sensory aids; however, uptake to sensory care is low. Notably, less than 20% of persons with hearing impairment have hearing aids and over 55% of Medicare Beneficiaries with reported vision problems have not had an eye examination in the prior year. Affordability and access may contribute to lack of sensory care uptake as Medicare explicitly excludes coverage of vision and hearing services. In this symposium, we will review current and new evidence for whether sensory loss affects health care outcomes, including satisfaction with care, incident delirium during hospitalization, navigation of Medicare, and present data on how persons with sensory loss are more likely to delay their care independent of cost and insurance factors suggesting fundamental changes in health care system interaction. We will place these results within the context of current national quality care and policy initiatives and review methods to address sensory loss.

